# Impairment of Interrelated Iron- and Copper Homeostatic Mechanisms in Brain Contributes to the Pathogenesis of Neurodegenerative Disorders

**DOI:** 10.3389/fphar.2012.00169

**Published:** 2012-09-25

**Authors:** Tina Skjørringe, Lisbeth Birk Møller, Torben Moos

**Affiliations:** ^1^Section of Neurobiology, Biomedicine Group, Institute of Medicine and Health Technology, Aalborg UniversityAalborg, Denmark; ^2^Center for Applied Human Molecular Genetics, Department of Kennedy Centre, Copenhagen University HospitalRigshospitalet, Copenhagen, Denmark

**Keywords:** blood-brain barrier, cerebrospinal fluid, copper, homeostasis, iron, neurodegenerative disorders

## Abstract

Iron and copper are important co-factors for a number of enzymes in the brain, including enzymes involved in neurotransmitter synthesis and myelin formation. Both shortage and an excess of iron or copper will affect the brain. The transport of iron and copper into the brain from the circulation is strictly regulated, and concordantly protective barriers, i.e., the blood-brain barrier (BBB) and the blood-cerebrospinal fluid (CSF) barrier (BCB) have evolved to separate the brain environment from the circulation. The uptake mechanisms of the two metals interact. Both iron deficiency and overload lead to altered copper homeostasis in the brain. Similarly, changes in dietary copper affect the brain iron homeostasis. Moreover, the uptake routes of iron and copper overlap each other which affect the interplay between the concentrations of the two metals in the brain. The divalent metal transporter-1 (DMT1) is involved in the uptake of both iron and copper. Furthermore, copper is an essential co-factor in numerous proteins that are vital for iron homeostasis and affects the binding of iron-response proteins to iron-response elements in the mRNA of the transferrin receptor, DMT1, and ferroportin, all highly involved in iron transport. Iron and copper are mainly taken up at the BBB, but the BCB also plays a vital role in the homeostasis of the two metals, in terms of sequestering, uptake, and efflux of iron and copper from the brain. Inside the brain, iron and copper are taken up by neurons and glia cells that express various transporters.

## Introduction

The transition metals, iron and copper are co-factors for a variety of proteins that are vital for the normal function of cells. In the brain, iron and copper are important co-factors for enzymes involved in, e.g., neurotransmitter synthesis and myelin formation (Gaggelli et al., 2006; Lutsenko et al., 2010). When in excess, they both participate in the Fenton reaction – a series of chemical reactions initiated by transition metals and hydrogen peroxide – which leads to the formation of unstable free radicals that significantly affect DNA, proteins, and lipids and cause deleterious damage to the cells (Rivera-Mancia et al., 2010). Therefore a strict regulation of these metals is vital for normal cellular function and survival.

In biological systems, iron mainly exists in two ionic forms, i.e., ferrous iron (Fe^2+^) and ferric iron (Fe^3+^). Outside the cell, iron is in its oxidized, almost insoluble Fe^3+^ state and is virtually completely bound to the liver-derived protein transferrin (Table 1). Intracellularly, iron mainly occurs as Fe^2+^. Most inorganic iron in the diet is in the Fe^3+^ form and must be reduced to Fe^2+^ before it can be taken up by the divalent metal transporter-1 (DMT1) which is located in the apical membrane of gastrointestinal cells. This reduction is probably facilitated by duodenal cytochrome *b*, DcytB (McKie et al., 2001), and possibly other reductases like Steap2, which is abundantly expressed in the duodenum and localizes to the cell membrane (Ohgami et al., 2006). Also heme-bound iron enters enterocytes but by a mechanism that does not involve DMT1. The heme carrier protein 1 (HCP-1) is a likely candidate for heme-iron uptake in the intestine (Shayeghi et al., 2005; Latunde-Dada et al., 2006). Cellular heme-iron uptake is followed by heme-oxygenase-mediated degradation of heme and the release of Fe^2+^ (West and Oates, 2008). Fe^2+^ from either route is subsequently transported through the enterocytes by unknown mechanisms to the basolateral membrane to where it is probably exported to the circulation by ferroportin in conjunction with being reoxidized to Fe^3+^ by the copper-containing protein, hephestin (Frazer et al., 2001; Anderson et al., 2002). In the circulation, the iron binds to the liver-derived transferrin (apotransferrin) which is the main ferric iron-binding protein in plasma. Under normal physiological conditions, there is virtually no non-transferrin bound iron in the circulation, as the iron-binding capacity of transferrin is far from saturated. Transferrin bound iron in the blood plasma is taken up by cells that express transferrin receptor 1 located on the cell membrane followed by clathrin-mediated endocytosis (Moos and Morgan, 1998; Levy et al., 1999).

**Table 1:** **Transporters involved in transport of iron and copper, respectively, in different cell types**.

Ionic forms	Transport in intestine	Transport in the circulation	Transport in peripheral cells	Transport across the BBB	Transport across the BCB	Transport in neurons	Transport in astrocytes
**IRON**							
Fe^2+^ (intracellularly)	DMT1 (Mem)	Transferrin	TfR	TfR	TfR	TfR	DMT1 (Mem)
Fe^3+^ (extra-cellularly)	Ferroportin + HP		DMT1 (End)		DMT1 (End)	DMT1 (End)	Ferritin
	Haem-iron: HCP-1, not		Ferritin		DMT1 (Mem)	Ferritin	Ferroportin + GPI- anchored CP
	involving DMT1		Ferroportin + CP		Ferroportin + HP	Ferroportin + CP + HP	(No TfR expression)
**COPPER**							
Cu^+^ (intracellularly)	Ctr1	CP	Ctr1	Ctr1	Ctr1	Ctr1	Ctr1
Cu^2+^ (extra-celullarly)	DMT1 (Mem)	Albumin	ATOX1	ATOX1	DMT1 (Mem)	ATOX1	ATOX1
	ATP7A		ATP7A/ATP7B	ATP7A	ATOX1	ATP7A	ATP7A
					ATP7A	ATP7B	
					ATP7B		

*DMT1 (Mem), DMT1 located in the cell membrane; DMT1 (End), DMT1 located in endosomes; CP, ceruloplasmin; HP, hephestin; TfR, transferrin receptor. Underlined text represents molecules of interaction between iron and copper metabolism. See text for further details*.

Copper is also a redox-active transition metal which readily changes its oxidation state between Cu^+^ and Cu^2+^ (Sharp, 2004). Outside the cell, copper mainly appears as Cu^2+^, and intracellularly mainly as Cu^+^. The uptake of dietary copper in intestinal epithelial cells is carried out by the high-affinity copper transporter-1 (Ctr1) located apically in the plasma membrane. Prior to its import to the cell, the Cu^2+^ is reduced to Cu^+^. The reductase involved in this process is not yet identified, but it has been demonstrated that members of the Steap family are able to perform this reduction in cell cultures (Ohgami et al., 2006). DMT1 might also be involved in the transport of copper ions into enterocytes (Arredondo et al., 2003; Sharp, 2004), although the statement was recently questioned (Illing et al., 2012). Several *in vitro* studies have shown that DMT1 can transport copper in Caco-2 cells (Arredondo et al., 2003; Espinoza et al., 2012), but the significance of DMT1 for intestinal copper uptake *in vivo* remains to be established. However, DMT1 seems to be involved in copper metabolism *in vivo* as shown in the Belgrade rat, which carries a mutation in DMT1. The iron-deficient Belgrade rat is not compromised in copper status, which is in contrast to iron-deficient wild type rats, which show an increase in serum and hepatic copper levels (Jiang et al., 2011). This result indicates that DMT1 is to some extent involved in copper metabolism.

In contrast to iron, it is also possible that copper gets transported through enterocytes by passive diffusion (Varada et al., 1993). Inside cells, copper is bound to one of several copper chaperones (CCS, Cox17, Cox11, ScoI, ScoII, ATOX1; Huffman and O’Halloran, 2001; Puig and Thiele, 2002; Madsen and Gitlin, 2007; Lutsenko et al., 2010) or to storage and ligand molecules like metallothionein (MT) and glutathione (GSH), respectively. The P-type ATPase ATP7A mediates the basolateral release of copper from the enterocyte into the circulation. The copper chaperone ATOX1 is involved in the transport of copper to ATP7A inside the cell. In blood plasma, copper is mainly bound to ceruloplasmin, but also for instance to albumin (Table 1). Copper is then transported from the circulation into cells in its Cu^+^-form by Ctr1 in similarity with the transport into the intestinal epithelial cells, described above (Lee et al., 2002). Upon entering cells from the circulation the copper is – like in the enterocytes – bound to chaperones and storage molecules. Similarly to iron, the cytosolic concentration of unbound copper is very low (Rae et al., 1999; Huffman and O’Halloran, 2001).

**Figure 1:**
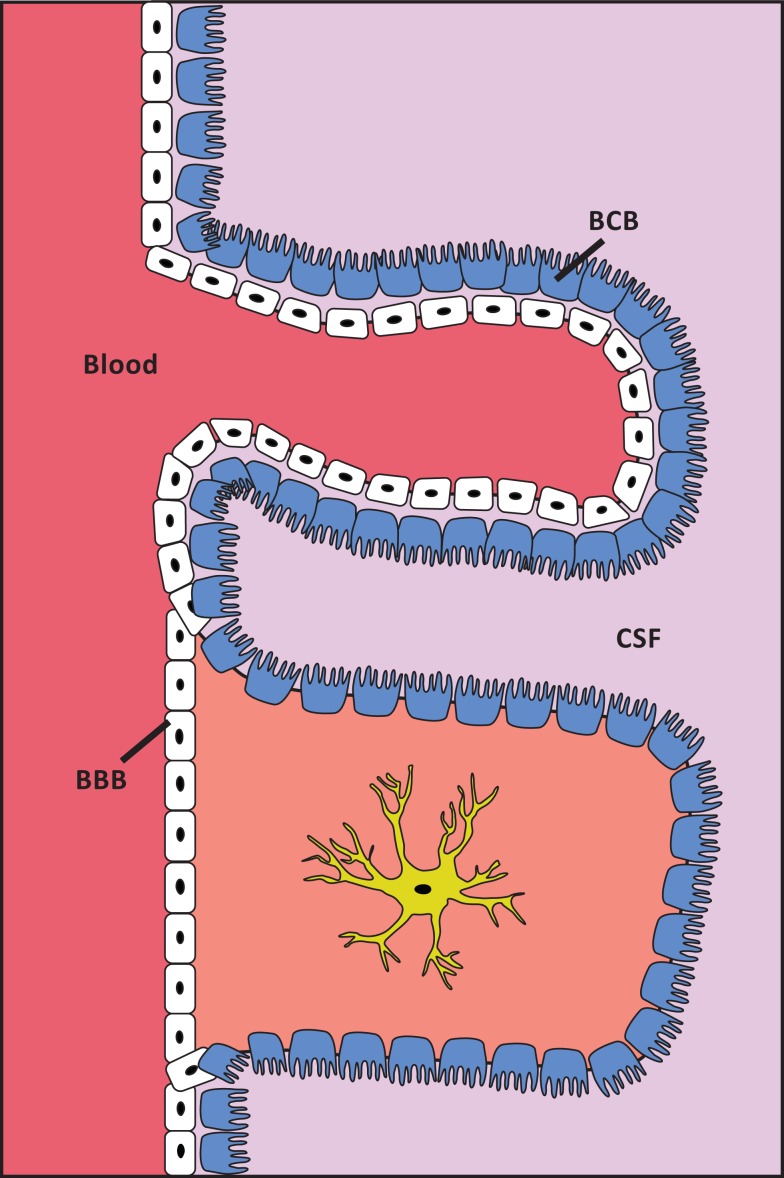
**Schematic presentation of the localization of the BBB and the BCB**. White cells are capillary endothelial cells. Blue cells are ependymal cells. The structure of cell layers in the choroid plexus/BCB is shown in the top of the figure. The structure of cell layers elsewhere in the brain/BBB is shown in the lower part of the figure. The capillary endothelial cells are tightly bound by tight junctions, except at the choroid plexus. In the choroid plexus the ependymal cells are, in contrast to elsewhere in the brain, tightly bound by tight junctions.

The transport of molecules from the circulation into the brain is strictly regulated, and is hindered by the protective barriers that separate the brain environment from the circulation (Figure 1). The blood-brain barrier (BBB) separates the blood circulation from the brain interstitial fluid, and the blood-cerebrospinal fluid (CSF) barrier (BCB) separates the blood from the CSF. The BBB consists of brain capillary endothelial cells that – in contrast to peripheral endothelial cells – are intimately connected by tight junctions and are non-fenestrated. Astrocytes and pericytes also contribute to the formation and maintenance of the BBB. The brain capillary endothelial cells are not uniform throughout the brain, as capillaries in the circumventricular organs and the choroid plexus contain fenestrated endothelial cells. The blood-CSF barrier is located between the blood and the choroid plexus. The epithelium of the choroid plexus is polarized with the basolateral side facing the blood, whereas the apical part contains microvilli that are in direct contact with the CSF. All solutes are transported through the blood-brain and blood-CSF barriers to the brain interstitial fluid and CSF, respectively. The cells denoting these barriers transport essential nutrients, metals, and pharmaceuticals into the brain in a controlled manner (Begley and Brightman, 2003; Abbott et al., 2006).

This review addresses the uptake and transport of iron and copper in the brain and discusses how these essential metals may play a role in the pathogenesis of brain diseases via their absence or their gradual accumulation due to hereditary or environmental dietary events. As the regulation of iron and copper transport in the brain strongly interact with each other, a separate section is devoted to how any imbalance of iron and copper homeostasis in the brain may affect their inter-relations and eventually affect neuronal functions.

## Regulation

The expression and activity of iron and copper transporters are regulated at different levels. Most iron transporters are regulated in response to iron levels in the cell by binding iron-regulatory protein 1 or 2 (herein collectively referred to as iron responsive proteins, IRPs) to the iron-response element (IRE) located in the UTR regions of the mRNA of these transporters. At high iron concentrations the IRPs binds iron, which changes the conformation or stability of IRPs and makes them unsuitable for binding to IRE. Ferroportin and ferritin have an IRE element in the 5′UTR of their mRNA, whereas the IRE element is located in the 3′UTR of the mRNA of the transferrin receptor and DMT1. Only two of four DMT1 isoforms have an IRE element. The binding of IRP to ferroportin and ferritin mRNAs mediates a repression of their translation, whereas the binding of IRP protects the mRNA of transferrin receptor and DMT1 from degradation (Wang and Pantopoulos, 2011). Furthermore, ferroportin expression is regulated by circulating hepcidin which induces its internalization (Nemeth et al., 2004). Hepcidin is also suggested to regulate DMT1 expression as DMT1 mRNA levels decrease in Caco-2 cells cultured in hepcidin-conditioned medium (Mena et al., 2006).

The general expression level of copper transporters occur independently of the cellular copper levels, however the subcellular localization of many transporters are affected by the copper state of the cells. Hence, at steady state levels, Ctr1 constitutively cycles between the plasma membrane and intracellular compartments, whereas at high concentrations Ctr1 is internalized at the cellular membrane and subsequently degraded (Petris et al., 2003). At steady state levels, ATP7A and ATP7B are mainly localized in the trans-Golgi network where they are responsible for incorporating copper into Cu-proenzymes. At high copper concentrations, the two proteins distribute to post-Golgi vesicles and even to the cellular membrane in order to facilitate the export of copper from the cell. The relocalization of ATP7A and ATP7B is reversible and does not require *de novo* protein synthesis (Petris et al., 1996; Hung et al., 1997; Roelofsen et al., 2000; van den Berghe and Klomp, 2010).

## Iron Transport and Homeostasis in the Brain

Like most other cell types, brain capillary endothelial cells take up iron by binding holotransferrin to the transferrin receptor, followed by endocytosis of the holotransferrin-transferrin receptor complex (Moos and Morgan, 1998; Levy et al., 1999). In other cell types, Fe^3+^ is detached from the receptor-ligand complex within the endosome, is reduced to Fe^2+^, and is pumped out into the cytosol by DMT1. Subsequently, the iron concentration is instantly regulated by three different processes, (1) the incorporation into organic molecules in its ferrous form, (2) storage as ferritin-iron subsequent to its re-oxidization, or (3) ferroportin-mediated export from the cell. However, presumably neither DMT1 nor ferroportin are expressed in brain capillary endothelial cells as demonstrated by protein blotting, immunohistochemistry and *in situ* hybridization on brains from adult rats and mice, or from iron-deficient rats (Gunshin et al., 1997; Moos and Morgan, 2004; Moos et al., 2006; Rouault et al., 2009). Notably, some controversy exist on the expression of DMT1 and ferroportin in brain capillary endothelial cells (Burdo et al., 2001; Yang et al., 2011; McCarthy and Kosman, 2012).

**Figure 2:**
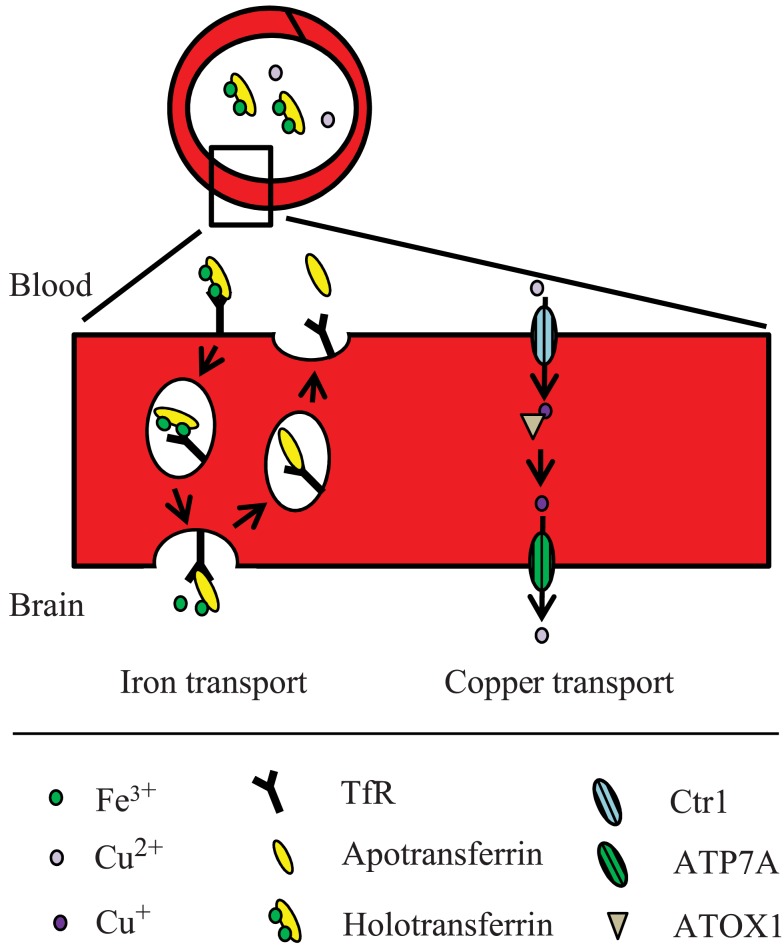
**Proposed model for iron and copper transport across the BBB**. The mechanisms are described in the text.

Hence, presumably a different route from the one normally employed during holotransferrin-mediated uptake could occur at the BBB. One hypothesis is that iron is transcytosed through the cells with little or no iron being pumped out of the trafficking vesicles into the cytosol (Moos et al., 2007; Figure 2). Instead, the vesicle carrying the transferrin-iron complex may dock at the abluminal surface of the brain capillary endothelial cell where the complex is subjected to the local factors of the microenvironment such as ATP, nucleotides and citrate secreted from astrocytes, which circumscribe the abluminal part of brain capillary endothelial cells, leading to iron release (Morgan, 1977, 1979). The iron-free apotransferrin, which is not released from the transferrin receptor during the transcellular transfer, is recycled inside transferrin receptor-containing endosomes to the luminal side of the brain capillary endothelial cells, from where it is returned to the circulation (Moos and Morgan, 2004).

The uptake-level of iron by the brain remains very constant, unless the concentration of iron in the brain is reduced. Should that happen, receptor internalization by brain capillary endothelial cells is significantly elevated resulting in an increased extraction of iron from blood plasma and transport into the brain (Monnot et al., 2011). Conversely, if iron levels in the blood plasma are raised, the brain capillary endothelial cells cease to take up iron (Morgan, 1996). In turn, these operating mechanisms at the BBB strongly protect the brain from being affected by fluctuations in systemic iron, and any disturbance of systemic iron homeostasis will have minimal effect on the iron levels in the CNS (Moos and Morgan, 2004).

Inside the brain, iron is taken up by neurons and glia cells by putatively different mechanisms. A recent study demonstrated that neurons in primary culture do not take up non-transferrin bound iron by a mechanism that involves binding to DMT1 (Pelizzoni et al., 2012). Neurons contain transferrin receptors and DMT1 located in endosomes, and most likely acquire iron by classical holotransferrin internalization followed by DMT1-mediated pumping of iron into the cytosol. In contrast, astrocytes and oligodendrocytes are likely to take up non-transferrin bound iron as they are devoid of transferrin receptors, even at substantial iron deficiency levels, where neurons increase their expression of transferrin receptors significantly (Moos et al., 1998). There are at least two distinct routes for uptake of non-transferrin bound iron in astrocytes, and one of these is DMT1-independent (Lane et al., 2010). The details of these routes are unknown. However, the metal transporters Zip 8 and Zip 14 may be involved as they have been shown to transport several divalent metal ions including iron (Fe^2+^) but not copper-ions (Pinilla-Tenas et al., 2011; Jenkitkasemwong et al., 2012). mRNA from both Zip 8 and Zip 14 has been identified in tissue from mouse brain (Girijashanker et al., 2008).

Judging from the rather heterogeneous distribution of ferritin in the brain, residual iron is stored in neurons and some glial cell types (Benkovic and Connor, 1993). Hence, some neuronal nuclei, e.g., hippocampal, hypothalamic, and mesencephalic neurons express ferritin, whereas a number of neuronal nuclei are devoid of ferritin-containing neurons (Hansen et al., 1999). This is however not true for the aged brain in which neuronal iron and ferritin levels increase (Benkovic and Connor, 1993). Non-neuronal cells, in particular microglia and oligodendrocytes, also increase their content of iron and subsequently express more ferritin with age (Benkovic and Connor, 1993).

**Figure 3:**
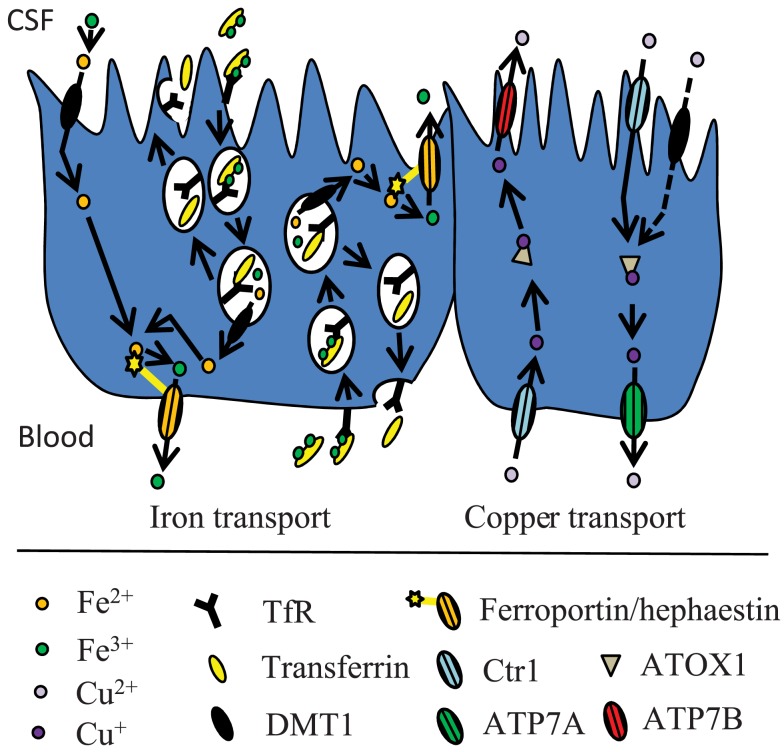
**Proposed model for iron and copper transport across the BCB**. The mechanisms are described in the text.

Extracellular iron in the brain is supposedly handled via its binding to transferrin and low-molecular weight substances like ATP, nucleotides and citrate. Extracellular transferrin is quite scarce in the brain and is mainly derived from synthesis and secretion by the choroid plexus and transcytosis of liver-derived transferrin at the choroid plexus (Moos et al., 2007; Rouault et al., 2009). Furthermore, the choroid plexus epithelial cells play a role in brain iron homeostasis, which is supported by the expression analyses that demonstrated the presence of various iron transporters such as transferrin receptor, DMT1, and ferroportin (Rouault et al., 2009; Figure 3). The relative contribution to the total iron transport into the brain mediated by the choroid plexus is however difficult to determine. Theoretically its contribution could be substantial, as the surface area of the choroid plexuses is at least half the area of the BBB (Keep and Jones, 1990), and the regional blood flow through their capillaries is approximately fivefold higher compared to many other regions in the brain (Maktabi et al., 1990). The choroid plexus epithelial cells have apical microvilli, suggesting that these cells play an absorptive role, which might be involved in iron transport from the CSF to the blood. This theory is supported by a study of rat primary choroid epithelial cells in a two-chambered Transwell system (Wang et al., 2008), where free iron was transported across the cells from the apical to the basolateral side. Apically located DMT1 played a major role in this process. The iron-transporting proteins expressed by the choroid plexus might also play a significant role in managing cellular iron utilized by the epithelial cells themselves.

## Copper Transport and Homeostasis in the Brain

The concentration of copper in the brain is higher than in other organs except for the liver (Lech and Sadlik, 2007). The level of copper uptake varies in different regions of the brain, e.g., copper uptake is significantly higher in the hippocampus than in the cerebellum (Choi and Zheng, 2009). This may reflect differences in regional blood flow, capillary density, or the abundance of copper transporter expression. The bulk of copper transport into the brain is thought to occur at the BBB, but the choroid plexus is also capable of transporting copper (Monnot et al., 2011; Figures 2 and 3). Using choroid epithelial cells, Z310, as an *in vitro* model of the BCB, siRNA specifically targeting the mRNAs of Ctr1, DMT1, ATOX1, and ATP7A respectively revealed that all of these proteins presumably are involved in copper transport across the choroid plexus (Monnot et al., 2012).

It is believed that copper is transported into the brain as a free ion. Using the brain perfusion technique, it was shown that the uptake of free copper (CuCl_2_) into brain capillaries, the choroid plexus and the CSF is much higher than the uptake of Cu-albumin or Cu-ceruloplasmin. The highest uptake from the blood was observed in the choroid plexus (Choi and Zheng, 2009). However, the further transport of copper into the CSF was almost 1200-fold slower, indicating that the choroid plexus sequesters the metal and strictly regulates its further movement into the brain (Choi and Zheng, 2009).

Whereas DMT1 presumably is absent from the brain capillary endothelial cells, there is plenty of Ctr1 which is assumed to play an important role in copper transport from the blood into the brain across the brain capillary endothelial cells. This notion is supported by studies on the Ctr1^+/−^ mouse, which – when compared to controls – has an approximately 50% lower expression of Ctr1 and a concurrent 50% lower copper transport level into the brain (Lee et al., 2001). Ctr1 is also widely expressed in the choroid plexus, and even more than in the brain capillary endothelial cells. Furthermore, the amount of Ctr1 is increased in the choroid plexus in copper-deficient mice (Kuo et al., 2006). The Ctr1 on the apical surface of the choroid plexus epithelial cells is believed to play an important role in copper transport from the CSF out of the brain.

ATP7A has been shown to facilitate copper transport across brain endothelial cells *in vitro*, and is essential for transporting copper further into the brain (Qian et al., 1998). This fits well with the fact that the brindled mutant mouse (a mouse model for Menkes disease) with a mutated ATP7A gene, has a higher than normal copper accumulation in brain capillaries combined with copper deficiency in the brain (Yoshimura et al., 1995). The related P-type ATPase ATP7B is also expressed in the brain, but not in brain capillary endothelial cells, and is therefore not believed to play a major role in copper transport into the brain (Qian et al., 1998; Barnes et al., 2005). ATP7B is however expressed in the choroid plexus, although at 14-fold lower levels than those of ATP7A (Choudhuri et al., 2003). Characteristically, ATP7B traffics to sub-luminal vesicles, but not to basolateral membranes, and is presumably involved in copper transport from the blood to the CSF.

Inside the brain, copper is utilized by a large number of important neuronal enzymes such as cytochrome *c* oxidase, Cu, Zn – dependent superoxide dismutase, peptidylglycine alpha amidating monooxygenase, dopamine beta monooxygenase, tyrosinase, and lysyl oxidase (Lutsenko et al., 2010). Furthermore, copper exerts antagonistic-like action on glutamatergic *N*-methyl d-aspartate (NMDA) receptors. In cultured hippocampal neurons, addition of copper leads to a rapid and reversible trafficking of ATP7A to neuronal processes regardless of the intracellular copper concentration. The copper acts specifically on NMDA receptors and leads to a rapid release of copper from the hippocampal neurons (Schlief and Gitlin, 2006). In contrast, a simultaneous activation of NMDA receptors does not lead to release, but to an enhanced uptake of ferrous iron in cell cultures (Cheah et al., 2006).

Metallothionein is strongly expressed in choroid plexus epithelial cells, suggesting that these cells serve as a reservoir for copper (Penkowa et al., 1999). An *in vitro* two-chamber model of the BCB revealed that the choroid plexus epithelial cells may play a key role in copper homeostasis in the brain by expelling copper from the CSF to the blood side; the main direction of the copper transport went from the apical side to the basolateral side of the epithelial cells (Monnot et al., 2011). Interestingly, the choroid plexus exhibited the highest capacity for copper uptake from the blood when compared to brain capillaries, brain parenchyma, and CSF *in vivo*. The transport is however 1200-fold slower from the choroid plexus into the CSF (Choi and Zheng, 2009). Thus, the blood-CSF barrier might play a considerable role in the regulation of copper by maintaining copper at a certain level, by sequestering copper from the blood and exporting excess copper out of the CNS back to the blood.

## Iron-Related Neuronal Pathologies

Characteristically for transition metals such as iron and copper, both their absence and excess affect organs throughout the body, including the brain.

Deficiency in iron mediates changes in the expression of a number of iron-related genes and their resulting proteins in the brain leading to, e.g., a rise in neuronal transferrin receptors and a decrease in neuronal ferritin, whereas the accumulation of iron by the brain reverses their expression (Hansen et al., 1999). Brain iron accumulation occurs in many chronic neurodegenerative diseases like Parkinson’s disease and Huntington’s disease. Excess iron is also found in the brain of patients with rare mutational diseases, where genes involved in the management of iron are affected.

### Brain pathology resulting from deficiency in brain iron

Iron deficiency in the brain leads to hypomyelination (Beard and Connor, 2003), abnormal cognition, changes in neurotransmitter metabolism, and impaired motor function (Kwik-Uribe et al., 2000a; Grantham-McGregor and Ani, 2001; Beard et al., 2006). If the iron deficiency occurs during early development, lifelong cognitive and motor impairment are observed in both rodents and humans (Kwik-Uribe et al., 2000a; Siddappa et al., 2004; DeBoer et al., 2005; Carlson et al., 2009). These impairments persists even after a complete iron repletion (Felt and Lozoff, 1996; Kwik-Uribe et al., 2000b; DeBoer et al., 2005; Felt et al., 2006; Lozoff et al., 2006). The adverse effects affect both brain morphology and development. For instance the Slc11a2*^hipp/hipp^* mouse, which has a conditional knock-out in the DMT1 gene in the hippocampus that results in regional iron deficiency, had abnormal hippocampal morphology and slower learning abilities (Carlson et al., 2009). A different rodent model showed that iron deficiency anemia during gestation and early postnatal life was accompanied by an altered expression of genes involved in the regulation of synaptic activity and apical dendritic growth in the hippocampus (Jorgenson et al., 2003, 2005).

Postnatal P15 (P) rats with neonatal iron deficiency exhibited an altered expression in six of seven genes implicated in Alzheimer’s disease pathogenesis (Carlson et al., 2008). The same seven genes were upregulated in the Slc11a2*^hipp/hipp^* mouse from P5 to P25, but returned to normal levels by P25 (Carlson et al., 2008). An upregulation of these genes during development may however lead to a higher expression again in adulthood. This phenomenon was observed in another study (Basha et al., 2005) where the upregulation of the β-amyloid protein precursor (*app*) gene – one of the tested Alzheimer’s disease genes – was observed in the cortex of P15 rats after exposure to lead during the early postnatal period. Although the expression of *app* returned to normal in early adulthood, the increased expression was reactivated in senescence without re-exposure to the toxin. Hence, a deregulation of expression during development due to brain iron deficiency may affect gene expression and pathology later in life.

### Inherent human diseases accompanied by brain pathology and iron accumulation

Iron accumulation is directly involved in the brain pathology of some inherent diseases that directly affects the cellular handling of iron by neurons. One example is neuroferritinopathy, which is an autosomal dominant disease that results in extrapyramidal symptoms due to mutation in the gene encoding the ferritin light (L) chain (Dusek et al., 2012; Keogh et al., 2012). Ferritin is an iron storage complex composed of 24 protein subunits consisting of heavy (H) chains and light (L) chains. The L-chains are dominant in ferritin in organs that store iron, where it is responsible for iron nucleation into the safe ferric state. The H-chains are dominant in ferritin in organs with a high iron metabolism (Koziorowski et al., 2007). The composition of the two subunits in ferritin is implicated in keeping the balance between bound and labile iron. In the brain, neurons mostly contain H-rich ferritin, whereas microglia mostly contain L-rich ferritin (Connor et al., 1994). Patients with neuroferritinopathy have abundant iron accumulation in the brain and neuronal loss that causes involuntary movements, dystonia, spasticity, and rigidity. However, the patients display no systemic abnormality, except for a decreased level of serum ferritin (Curtis et al., 2001). It has been suggested that the mutations in L-ferritin impair the ferritin assembly and result in a loss of iron storage capacity within brain cells and a subsequent iron-mediated cell injury (Levi et al., 2005).

Another example is panthothenate kinase-associated neurodegeneration (PKAN; formerly Hallervorden–Spatz disease), which is characterized by excessive iron accumulation in the brain accompanied by neuronal loss (Zhou et al., 2001). The accumulation of iron in PKAN is thought to be secondary to a mutation in the gene encoding the enzyme pantothenate kinase 2. This mutation leads to the accumulation of cysteine residues that – because of their high-affinity for divalent metal ions – may cause accumulation of iron in neurons (Zhou et al., 2001).

A more recently identified mutation in the PLA2G6 gene that encodes a calcium-independent phospholipase A2 [iPLA(2)beta] also leads to progressive iron accumulation in the brain accompanied by neuronal dystrophy and cell loss (Gregory et al., 2008). Deletion of the gene in an experimental model leads to an iPLA(2)beta-loss-of-function, to severe neuronal dystrophy, and to brain iron accumulation (Malik et al., 2008).

### Iron accumulation in neurodegenerative diseases

Iron accumulates in brains of patients with Parkinson’s disease (Dexter et al., 1989, 1991). Similar observations have been made in patients suffering from other neurodegenerative diseases, e.g., Alzheimer’s disease and Huntington’s disease (Moos and Morgan, 2004). However, the association between iron accumulation and neurodegeneration has received most attention in an attempt to explain iron’s contribution to the pathogenesis of Parkinson’s disease. Experimental studies have confirmed that chronic degeneration in the brain is accompanied by the accumulation of iron (Sastry and Arendash, 1995). Although there is a clear indication that iron plays a significant role in neuronal cell death in patients suffering from neurodegenerative diseases, a direct connection to iron accumulation in the relevant brain regions has never been demonstrated directly. However, data from patients with hemochromatosis clearly indicate that iron deposition in non-neuronal organs is harmful to cells and leads to disease (Rouault, 2001). Experimental studies on cultured neurons have also provided evidence that iron damages neurons in a dose-responsive manner (Ostrerova-Golts et al., 2000), hence clearly suggesting that the exposure to excess iron is harmful to neurons. Apart from the directly intoxicated neurons, iron can propagate an oligomer-formation of alpha synuclein that leads to a gradual intoxication of alpha synuclein-containing aggregates.

Parkinson’s disease occurs as a result of dopaminergic neuronal death in the substantia nigra pars compacta. Excess labile iron that can participate in the Fenton reaction, is thought to contribute heavily to oxidative stress and subsequent degeneration among dopaminergic neurons (Halliwell, 1992). Many Parkinson’s disease patients have an – as yet unexplained – increased level of total iron in the substantia nigra as compared to age-matched controls (Sofic et al., 1988; Dexter et al., 1991; Griffiths and Crossman, 1993). The (+IRE) isoforms of DMT1 has been shown to be upregulated in Parkinson’s disease patients (Salazar et al., 2008). DMT1 may play a critical role in iron accumulation and in the pathogenesis of Parkinson’s disease as macrocytic mice (*mk/mk*) and Belgrade rats, which carry the same mutation in DMT1 that impairs iron export, are partially protected against parkinsonism-inducing neurotoxins (Salazar et al., 2008). Interestingly Parkin, a protein component of a multiprotein E3 ubiquitin ligase complex, which in its mutated form cause autosomal recessive juvenile Parkinson’s disease, is a regulator of expression of the 1B isoforms of DMT1 (Roth et al., 2010). Furthermore, iron could accumulate in neurons because of their decreasing ability to reuse the iron, thereby leading to their higher need for iron uptake (Moos et al., 2007). A decreasing ferroxidase activity mediated by ceruloplasmin in the Parkinson’s disease brain could result in a lower capacity for cellular iron export (Olivieri et al., 2011). Likewise, iron accumulation may also originate from iron-containing monocytes and macrophages that continuously migrate into the affected substantia nigra.

It should also be mentioned that the increase in brain iron does not always apply to Parkinson’s disease patients (Uitti et al., 1989; Galazka-Friedman et al., 1996; Loeffler et al., 1996). This could be due to the fact that the pool of labile iron stored in ferritin – and therefore available for the Fenton reaction – may vary, and may evoke oxidative damage in neurons even though the level of total iron is not raised. Several studies have demonstrated a decreased level of L-chain-ferritin in the substantia nigra of Parkinson’s disease patients compared to that of controls (Connor et al., 1994; Koziorowski et al., 2007). This decreased level could lead to an increased efflux of iron from the ferritin complex into the remaining cytosol as described in patients with neuroferritinopathy (see above), which in turn could lead to higher levels of free labile iron. Concurrently, a statistically significant twofold higher reactive oxygen species (ROS) activity was seen in substantia nigra tissue of Parkinson’s disease patients as compared to controls, notably without differences in total iron in the substantia nigra between the two groups (Wypijewska et al., 2010). Also ferritin accumulation has been reported in some – but not all – Parkinson’s disease patients (Dexter et al., 1991; Connor et al., 1995; Faucheux et al., 2002; Bartzokis et al., 2004). Chronic ferritin elevation has been shown to result in progressive age-related neurodegeneration of midbrain dopamine containing neurons (Kaur et al., 2007). Thus several paths may lead to the same result of iron mediated oxidative stress in Parkinson’s disease.

Alzheimer’s disease is accompanied by accumulation of iron in addition to zinc in β-amyloid (Aβ) – containing plaques. The aggregation of Aβ is induced by Zn^2+^. Aβ is derived from the amyloid protein precursor protein APP, which has recently been shown to be able to catalyze the binding of iron with transferrin and to interact with ferroportin (Duce et al., 2010). APP null (*app^−/−^*) mice suffer from iron accumulation and oxidative stress in cortical neurons, and accumulation of iron is comparable with that occurring in astrocytes in ceruloplasmin null (*cp ^−/−^*) mice. Zink-ions (Zn^2+^) inhibit the ferroxidase activity of APP and abnormal exchange of cortical zinc may link APP to iron accumulation in Alzheimer’s disease (Duce et al., 2010).

## Copper-Related Neuronal Pathologies

Copper is essential for normal CNS function. It is required as co-factor for dopamine beta monooxygenase, peptidylglycine alpha amidating monooxygenase, superoxide dismutase, and a large number of other enzymes. Furthermore it is required for mitochondrial respiration and oxidation of Fe^2+^ leaving the brain vulnerable in case it gets deprived in copper. On the other hand too much copper is toxic. Brain copper accumulation observed in Wilson disease results in parkinsonian symptoms such as dystonia and dysarthria.

### Impact of developmental copper-deficiencies on brain function

An interrupted copper homeostasis in the brain has severe effects. For instance, cerebral copper deficiency is associated with decreased neuronal cytochrome *c* oxidase activity that consequently alters mitochondrial function and brain energy metabolism (Gallagher et al., 1956). The development of the brain is also likely to become affected due to a decreased amount of dopamine-β-monooxygenase which leads to a lower level of noradrenaline synthesis (Prohaska and Wells, 1974). Severely copper-deficient mice, with a knock-out of the Ctr1 gene, exhibit growth, and viability defects which can be partially rescued by postnatal copper administration (Nose et al., 2006). The neurological effects of copper deficiency during perinatal life strongly depends on the timing of the deficiency, suggesting a critical period for brain copper acquisition (Prohaska and Brokate, 2001). An increase in the copper concentration between P7 and P14 was observed in the striatum, thalamus, and superior colliculus in rats (Tarohda et al., 2005). Copper deficiency in adult humans leads to ascending sensory tract dysfunction and neurodegeneration of the sensory dorsal column resulting in myelopathy accompanied by spasticity and sensory ataxia. Copper supplementation prevents further neurologic deterioration in such patients, but the degree of actual improvement is variable (Kumar et al., 2004; Prodan et al., 2006).

### Inherited diseases that lead to impaired management of copper in the brain

Menkes disease is caused by a mutation in the gene encoding the copper transporter ATP7A that results in severe copper deficiency in the brain. Affected individuals display psychomotor deterioration, neuronal loss, demyelination, and neurodegeneration in the gray matter of the brain (Okeda et al., 1991; Kaler, 1998; Kodama et al., 1999) and individuals with the classical severe form of Menkes disease die within the first 3 years of life. ATP7A is required to facilitate copper transport across the BBB (Qian et al., 1998), and the neurological characteristics of Menkes disease are present in early infancy and emphasize a critical role of copper in neuronal development. In Menkes disease patients with some residual and functional ATP7A, less neurological pathology is observed. In these patients copper therapy can improve the neurological symptoms (Kaler et al., 1995; Christodoulou et al., 1998; Moller et al., 2000).

Wilson disease is caused by a mutation in the gene encoding the copper transporter, ATP7B, which is primarily expressed in the liver, but also in the brain (Saito et al., 1999). The main symptoms of Wilson disease are hepatic copper accumulation, copper-mediated liver damage, leakage of copper into the plasma, and subsequent copper-overload in all tissues, including the brain (Gitlin, 2003; Tao and Gitlin, 2003). Brain copper accumulation leads to symptoms such as dystonia, psychiatric symptoms of depression, cognitive deterioration, personality change, schizophrenia, psychosis, and Parkinsonian symptoms (Oder et al., 1991; Ferenci, 2004). Almost half of all Wilson disease patients display symptoms of neuropsychiatric illness (Gollan and Gollan, 1998). However, the symptoms can be reversed by the administration of copper-chelating agents to the Wilson disease patient (Brewer et al., 2003, 2006). ATP7B is important for the delivery of copper to ceruloplasmin in the liver. The function of ATP7B in the brain is unknown, but it can be hypothesized that ATP7B also is involved in the delivery of copper to ceruloplasmin in the brain (see below).

Aceruloplasminemia is an autosomal recessive disorder of iron metabolism, caused by loss-of-function mutations in the gene encoding ceruloplasmin and with an adult-onset. Ceruloplasmin is a ferroxidase, which oxidizes ferrous iron to ferric iron, and a trinuclear cluster of copper is essential for the activity of ceruloplasmin. Ceruloplasmin is mainly expressed in the liver, but also in cells in the brain such as astrocytes and epithelial cells in the choroid plexus (Klomp and Gitlin, 1996; Klomp et al., 1996). The disease results in systemic symptoms such as decreased serum iron levels, elevated serum ferritin, anemia, and increased levels of non-transferrin bound iron, and neurological symptoms, such as progressive dementia, dystonia, neuronal cell loss, and excess iron accumulation in glia and neurons (Logan et al., 1994; Morita et al., 1995; Oide et al., 2006). The ceruloplasmin form expressed in astrocytes is attached to the cell surface by a glycosyl-phosphatidylinositol (GPI) anchor (Patel and David, 1997). Astrocytes in ceruloplasmin-deficient mice have a decreased ability to release iron from neurons (Jeong and David, 2003). The mechanism behind this effect is unknown, but might be due to a transfer of the GPI-anchored ceruloplasmin from the astrocytes to the neurons (Jeong and David, 2003). Accordingly, aceruloplasminemia leads to accumulation of iron in the brain, which explains many of the neurological symptoms mentioned above.

### Neurodegenerative disorders associated with impaired copper metabolism in the brain

Alzheimer’s disease is characterized by progressive neuronal degeneration, memory impairment, dementia, progressive decline in cognitive function, and volume loss in the telencephalon. It also leads to the formation of senile plaques that mainly consist of the amyloid beta peptides (generated from APP) and the formation of neurofibrillary tangles from abnormal tau proteins (Caselli et al., 2006; Kelley and Petersen, 2007). The involvement of copper in Alzheimer’s disease is not clear, as some studies show elevated serum-copper levels in Alzheimer’s disease patients (Squitti et al., 2006; Arnal et al., 2010), whereas others fail to find differences in copper levels when compared to controls (Ozcankaya and Delibas, 2002; Gerhardsson et al., 2008). Some studies even find decreased levels of copper in blood samples of Alzheimer’s disease patients (Brewer et al., 2010; Vural et al., 2010). A meta-analysis study performed on studies carried out from 1983 to 2010 showed that Alzheimer’s disease patients (761 individuals) generally have slightly higher levels of serum-copper than healthy controls (664 individuals; Bucossi et al., 2011b). However, no difference was observed in CSF-copper levels (116 Alzheimer’s disease patients and 126 healthy controls). Notably, both the aggregation and neurotoxicity of Aβ depend on the presence of copper. Furthermore, APP plays a role in regulation of cellular copper export. Several studies have shown that over-expression of APP leads to decreased copper in yeast (Treiber et al., 2004) and in brains of mice (Maynard et al., 2002). That copper metabolism is involved in the pathogenesis of Alzheimer’s disease is however, emphasized as several polymorphisms in the gene encoding the copper transporter ATP7B seem to be linked to the development of Alzheimer’s disease. Several single nucleotide polymorphisms (SNPs) in the ATP7B gene have been shown to increase the relative risk of developing Alzheimer’s disease by up to 82% (Bucossi et al., 2011a, 2012).

Several studies have also demonstrated a connection between copper accumulation and the pathogenesis of Parkinson’s disease. The total copper content is reduced in the substantia nigra compacta and caudate nucleus in Parkinson’s disease patients (Dexter et al., 1989; Loeffler et al., 1996). The administration of copper is, to some extent, neuroprotective against dopaminergic degeneration.

## Interactions in the Handling of Iron and Copper in the Brain

### Interaction between copper and iron metabolism in dietary challenges

Many studies have demonstrated an interaction between copper and iron after challenges to their dietary availability levels (Prohaska and Wells, 1974; Penland and Prohaska, 2004). An overview of reported effects is shown in Table 2. Dietary deficiency of either copper or iron leads to anemia and a higher liver concentration of the other metal (Pyatskowit and Prohaska, 2008). Copper and iron interact at the molecular level, e.g., copper binds to and regulates the activity of the IRP, which is a regulator of expression of multiple proteins involved in iron metabolism as described previously. It is known that the level of iron regulates the binding capacity of the IRP to the IRE. Interestingly, copper also mediates decreased binding of IRE/IRP by direct binding of the metal to IRP at the site, which also binds iron (Oshiro et al., 2002).

**Table 2:** **Effect of high or low systemic levels of metal 1 on the level of metal 2 in the brain and on transporters of metal 2**.

Systemic level of metal 1	Effect on brain level of metal 2	Effect on transporters of metal 2
Iron ↓	Copper transport↑	ATOX1 ↑
	Copper in CSF ↑	ATP7A ↑
	Brain parenchyma↑ Choroid plexus↑	
Iron ↑	Copper transport ↓	DMT1 (+IRE)↓
	Copper in CSF ↓	TfR ↓
	Brain parenchyma↑ Choroid plexus↑	Ferroportin↑
Copper ↓	Iron ↓(short term)	Hephestin↓
	Iron ↑ (long term)	GPI-ceruloplasmin↓
Copper ↑	Iron ↓	DMT1 (+IRE)↓
		TfR ↓
		Ferroportin↑
Copper↑ + Iron ↑	Copper↑	
	Iron ↑	

*Metal 1 is iron or copper, respectively. Metal 2 is the opposite metal, copper or iron, respectively. TfR, transferrin receptor*.

The response of IRP to copper is similar to the response to iron (Garrick et al., 2003b). The effect of copper on the IRP/IRE binding is emphasized by an approximately 55% decreased mRNA expression of the transferrin receptor in response to high copper concentrations *in vitro* (Oshiro et al., 2002). Human PCL hepatoma cells were in this study exposed to 150 μM copper, which is higher than in normal human serum. However, in pathophysiological conditions levels of 200 μM serum-copper and higher has been reported (Song et al., 2009). Furthermore, studies of Caco-2 cells have shown that excess copper results in a decreased mRNA and protein expression of the DMT1-isoform containing an IRE, and an increased expression of the iron exporter ferroportin (Tennant et al., 2002). Concurrent with the increased expression of ferroportin, a 40% increase in iron efflux from the Caco-2 cells was seen (Tennant et al., 2002). Furthermore, both copper and iron are transported with high efficiency by DMT1 as shown in *in vitro* assays (Garrick et al., 2003a). Notably, it has been shown in knockdown studies on immortalized choroid cells (Z310), that Ctr1 is the main transporter of copper over DMT1 (Zheng et al., 2012). However, in cells where DMT1 is located to the cell membrane, as in duodenal enterocytes and epithelial cells of the choroid plexus, there is a coupling between copper and iron homeostasis.

The interaction between the copper and iron homeostasis apparatus has been demonstrated *in vitro* on choroid epithelial cells, BCB Z310. In response to iron deficiency, the expression of DMT1 mRNA as well as the dedicated copper transporters ATOX1 and ATP7A is upregulated, whereas there is no change in the expression levels of Ctr1 (Monnot et al., 2012). The same regulatory picture is seen *in vivo* in duodenum, where the expression of DMT1 and ATP7A, but not of Ctr1 or ATP7B, is strongly induced in rat duodenum in response to dietary iron deficiency; significantly higher liver-copper levels were additionally observed (Collins et al., 2005). Concordantly, iron-deficient rats display a significantly higher rate of copper clearance from the CSF as compared to controls and copper overloaded animals (Monnot et al., 2011). The effect of iron deficiency on copper export and ATP7A might be attributed to increased transcription levels. Recent results have shown that ATP7A is upregulated by the transcription factor hypoxia inducible factor (HIF2alpha) in response to iron deficiency in rat intestinal epithelia (Xie and Collins, 2011), which could also be the case in the brain.

### Effect of abnormal copper levels on the level of iron

Both copper deficiency and excess lead to altered iron homeostasis in the brain. For instance, in rats which are fed a copper-rich diet, the iron uptake in the brains of P15 and P63 rats is significantly lower than in controls (Crowe and Morgan, 1996). However, dual administration of iron and copper in the diet results in an increase in both metals in the brain (Crowe and Morgan, 1996).

Copper deficiency was shown to confer 49% reduction in plasma iron levels but unchanged brain iron levels in P50 rats after 30 days on a postnatal copper-deficient diet (Prohaska and Gybina, 2005). The low serum iron is probably due to impaired transport of iron from the duodenum to the circulation as a result of decreased activity of hephestin which is essential for the cellular iron export function of ferroportin (Umbreit, 2005). Iron uptake of the brain is supposedly independent of proenzymes containing copper, as described previously, which is concordant with no effect on brain iron levels in the copper-deficient rats. Brain copper levels were 37% reduced in the copper-deficient P50 rats and it can be speculated that over time the copper deficiency may lead to brain iron accumulation, as seen in pathological conditions like aceruloplasminemia, which has an adult-onset at the age of 45–55. The symptoms in aceruloplasminemia are caused by CNS iron accumulation due to defects in ceruloplasmin as described. GPI-anchored ceruloplasmin in astrocytes is vital for iron release from these iron storage cells. Copper deficiency has been shown to lead to lower GPI-anchored ceruloplasmin in spleen and liver of mice and rats, and the same may be the case for astrocytic GPI-anchored ceruloplasmin (Mostad and Prohaska, 2011). Increased turnover rate is suggested to be the reason for the decreased protein levels. Other copper-containing proteins are essential in iron metabolism and may lead to accumulation of iron due to copper deficiency, e.g., hephestin as the activity of this protein is vital for iron export via ferroportin. Ferroportin is abundantly expressed in the epithelial cells of the choroid plexus, which in turn is involved in iron transport out of the CNS and into the circulation. Hence, a decline in hephestin activity probably would contribute to iron accumulation in the brain during copper deficiency.

In rats perinatal copper deficiency leads to decreased plasma and brain iron levels (Prohaska and Gybina, 2005; Pyatskowit and Prohaska, 2008). Interestingly, varying results have been obtained in mice as one study finds unchanged plasma and brain iron levels when copper-deficient (Pyatskowit and Prohaska, 2008), whereas others report decreased plasma and iron levels in similarity to observations in the rat. Age and strain differences may explain the differing results. Human infants with copper deficiency have lower plasma iron levels and it is speculated that brain iron levels may also be low (Pyatskowit and Prohaska, 2008).

Prohaska and Gybina hypothesize that the observed iron deficiency pups from copper-deficient dams could be due to impaired transport of iron across the placenta, as it has been known that copper deficiency inhibits iron transport in placental BeWo cells *in vitro* (Danzeisen et al., 2002). It has been suggested that this phenomenon might be attributed to the lack of ferroxidase activity of a vital ceruloplasmin homolog which was identified in rat placenta cells and shown to have a lower level of activity and expression under copper-deficient conditions (Danzeisen et al., 2002). This ferroxidase may be zyklopen, which has high sequence identity to hephestin and ceruloplasmin but a distinct expression pattern. Zyklopen is expressed in placenta and in mammary glands, and the protein level is decreased in cellular copper deficiency (Chen et al., 2010). In the suckling pup, low iron levels are maintained as the iron content in milk is additionally lower in copper-deficient rats (Prohaska, 1989), which may also be due to altered activity of zyklopen. Thus, these results emphasize that adequate dietary copper in dams is necessary for adequate iron transfer to pups, which in turn, is crucial for brain development.

### Effect of abnormal iron levels on the level of copper

Both iron deficiency and overload lead to altered copper homeostasis in the brain. An *in vivo* study on adult rats showed that iron-deficient rats had a 92% increase in copper transport into the brain, and significantly elevated copper levels in the CSF, brain parenchyma, and choroid plexus, although the plasma copper levels were normal (Monnot et al., 2011). A significant increase in brain copper levels has also been shown in pups due to perinatal iron deficiency (Bastian et al., 2010). Iron-overloaded rats, in contrast, have been shown to have lower plasma copper levels, a 56% decrease in copper transport into the brain, and lower copper levels in the CSF. Interestingly, a modest increase of copper levels in brain parenchyma and choroid plexus was observed in the iron-overloaded animals (Monnot et al., 2011). The authors hypothesize that the observed increase in copper in the brain parenchyma and the choroid plexus in the iron-overloaded animals might be attributed to an increased demand for copper for the production of ceruloplasmin and hephestin to export excess iron. The higher transport of copper into the iron-deficient brain is not accompanied by a correspondingly higher uptake of ceruloplasmin (Crowe and Morgan, 1996).

## Outlook

The uptake of iron at the BBB is well documented when the availability of an appropriate diet varies, but its subsequent transport into the brain needs further attention. The putative absence of DMT1 in brain capillary endothelial cells is congruous with a transcytotic transport of holotransferrin, but confusingly there is no real evidence for the transport of transferrin across the BBB. Studies have failed to identify ferroportin in brain capillary endothelial cells. With regard to the transport of copper at the BBB, the evidence points to uptake by Ctr1. There is no luminally expressed DMT1 that could account for the uptake of low-molecular weight copper at the BBB. Copper circulates in the blood plasma bound to proteins like ceruloplasmin and albumin, but the mechanism by which these proteins could donate copper at the BBB is not clear.

The roles of transferrin and ceruloplasmin secreted by the choroid plexus epithelial cells in iron homeostasis, in terms of extracellular binding and donation to receptor-containing neurons and ferroxidase activity by astrocytes respectively, are still unexplained. More research is needed to elucidate the possible transport of transferrin and ceruloplasmin from the ventricular system to the brain interstitium.

Iron levels in the brain are clearly reflected in the expression patterns of transferrin receptors and ferritin in neurons. Iron is thought to contribute significantly to the deleterious outcome of acute and chronic lesions in the brain. The role of iron in these conditions is far from resolved. In spite of a consistent expression of ferroportin by neurons, there is no real information on whether neurons are capable of exporting iron to the cellular exterior. More knowledge about these processes will probably add significantly to the understanding of how iron can cause damage to neurons, and probably also to the identification of putative pharmaceutical targets to improve neuronal iron-handling. The molecules that transport copper inside the brain to uptake in neurons and glia are not well documented. In contrast to iron that accumulates in neurons in neurodegenerative diseases, the level of copper seems to decrease, suggesting that neurons are capable of excreting copper.

We are far from understanding how iron and copper together are handled by neurons and glia when changes in the availability of these metals occur. Experimental models using animals with loss-of-function mutations that affect single proteins will reveal more information on these matters.

Handling iron- or copper- deficiencies is manageable with oral or parenteral supplementations. More sophisticated strategies, e.g., genetic therapy will be needed to treat single-mutation diseases known to affect iron and copper levels in the brain. The use of chelators to treat conditions with robust accumulation of iron or copper will benefit from more knowledge on the site of action of the chelating drugs as the homeostasis in overload conditions often derives from metal-accumulation in single cell types in the brain.

## Conflict of Interest Statement

The authors declare that the research was conducted in the absence of any commercial or financial relationships that could be construed as a potential conflict of interest.

## Abbreviations

APP, amyloid precursor protein; BBB, blood-brain barrier; BCB, blood-CSF barrier; CSF, cerebrospinal fluid; Ctr1, copper transporter-1; Cu, copper; DMT1, divalent metal transporter-1; Fe, iron; Fe^2+^, ferrous iron; Fe^3+^, ferric iron; GSH, glutathione; IRE, iron-response element; IRP, iron-regulatory protein; MT, metallothionein; NMDA, *N*-methyl d-aspartate; P, postnatal.
